# The association of triglyceride–glucose index with major adverse cardiovascular and cerebrovascular events after acute myocardial infarction: a meta-analysis of cohort studies

**DOI:** 10.1038/s41387-024-00295-1

**Published:** 2024-06-06

**Authors:** Huiruo Liu, Liangshan Wang, Hong Wang, Xing Hao, Zhongtao Du, Chenglong Li, Xiaotong Hou

**Affiliations:** Centre for Cardiac Intensive Care, Beijing Institute of Heart, Lung and Blood Vessel Diseases, Beijing Anzhen Hospital, Capital Medical University, Beijing, China

**Keywords:** Cardiovascular diseases, Pathology, Obesity

## Abstract

**Background:**

Insulin resistance (IR) is indicated to be linked with adverse outcomes of acute myocardial infarction (AMI), for its pro-inflammatory and pro-thromboplastic function. The triglyceride-glucose (TyG) index is a newly developed substitute marker for IR. The aim of this pooled analysis was to provide a summary of the relationship of TyG index with occurrences of major adverse cardiovascular and cerebrovascular events (MACCEs) among populations suffering from AMI.

**Methods:**

Cohorts reporting multivariate-adjusted hazard ratios of TyG index with MACCEs or its independent events were identified through systematically searching PubMed, MEDLINE, Web of science, Embase and Cochrane databases. Results were combined using a random-effects model.

**Results:**

21 cohorts comprising 20403 individuals were included. Compared to individuals in the lowest TyG category, patients in the highest TyG category exhibited elevated risks of both MACCEs (*P* < 0.00001) and all-cause death (*P* < 0.00001). These findings were in line with the results as TyG analyzed as continuous variables (MACCEs: *P* = 0.006; all-cause death: *P* < 0.00001). Subgroup analysis demonstrated that diabetic status, type of AMI, nor the reperfusion therapy did not destruct this correlation (for subgroups, all *P* < 0.05).

**Conclusion:**

All these indicated that higher TyG index could potentially predict MACCEs and all-cause death in patients with AMI as an independent indicator.

## Introduction

Despite of substantial advances in the recent guideline-recommended treatment, acute myocardial infarction (AMI) remains extensively a major contributor to the mortality and morbidity of cardiovascular diseases globally [1, 2], non-negligibly accounting for recurrent major adverse cardiac and cerebral events (MACCEs) that has been recognizably correlated with worse prognosis in those with AMI [3]. Hence, early identification of potential risk factors for MACCEs in AMI population is imperative for optimal management to further reduce death and disability [4].

Insulin resistance (IR), a hallmark of metabolism disorder, not only functioned as a pivotal mechanism of AMI, but was also related to poorer prognosis [5, 6]. Pathologically, IR could result in vascular inflammation and abnormal coagulation, thereby promoting the accumulation of vascular lipids and thrombosis, ultimately accelerating arteriosclerosis and stenoses [7, 8]. Furthermore, IR hindered the uptake of glucose by ischemic myocardium and thus impeded their ability to generate energy through compensatory glycolysis, subsequently contributing to increased infarct zone and decreased myocardial contractility [5]. To date, population with type 2 diabetes mellitus whom prevalently coming up with IR, has been grouped as the extreme-risk crowd for recurrent MACCEs after AMI [9]. Classically, the hyper insulinemic euglycemic clamp was recognized the gold criterion for evaluating IR [10, 11]; nonetheless, it is too complex to popularize in large-scale clinical practice [12]. The triglyceride–glucose (TyG) index, which is derived from fasting plasma glucose and triglyceride levels, has been proposed as a dependable and accurate substitute marker for IR [13]. Accordingly, accumulating cohorts have been concerning the correlation of TyG index with MACCEs risks in AMI, whereas inconsistent conclusions were obtained: some showed that elevated TyG was apparently in relation to more frequent MACCEs, while others found this association to be non-significant [14–34]. Herein, for the first time, we aim to perform a pooled analysis evaluating the predictive value of baseline TyG index on MACCEs in AMI population.

## Methods

This review was reported in accordance with the PRISMA guidelines [35].

### Study search

We conducted a search in databases including PubMed, MEDLINE, Web of Science, Embase and Cochrane using a combination of terms: (1) “TyG index” OR “triglyceride-glucose index” OR “triglyceride glucose index” and (2) “heart infarction” OR “myocardial infarction” OR “cardiovascular stroke” OR “‘myocardial infarct” OR “myocardial infarct” OR “heart attack” OR “MINOCA” OR “cardiogenic shock”, from inception to October 18, 2023, without language restriction. Mannual hand-searching of reference lists from relevant trials and reviews was supplemented. The full search strategy is expanded in supplementary Table 1. Final inclusion was based on the consensus of two independent reviewers, or a third independent investigator would serve as the referee in case of disagreements.

### Study selection

Studies fulfilling the following criteria (according to the PECO framework) were included: (P) types of participants: consecutive adults diagnosed with AMI at baseline; (E) exposures and (C) comparators: high versus low TyG index; (O) outcomes: the primiary outcome was major adverse cardiac and cerebrovascular events (MACCEs), and the secondary outcomes were the independent events of MACCEs. TyG index was calculated via ‘ln [TG (mg/dl) * FBG (mg/dl)/2]’. The MACCEs was characterized as a combination of all-cause death, nonfatal stroke, nonfatal myocardial infarction, rehospitalization for HF, and revascularization. Exclusion criteria were set as: Cross-sectional studies for high bias risks; studies not reporting multivariable adjusted association for the correlation of TyG index with AMI prognosis; studies ongoing or in English. In case of overlap in the populations of different studies from the same registry or group, only the largest size was included.

### Data extraction and quality assessment

Two independent reviewers extracted and checked data. Data abstracted included: (a) first author’s name, and publication year; (b) research design, and follow-up duration; (c) patients characteristics, including study region, sample size, age, sex, type of AMI, diabetes proportion; (d) patterns of TyG index analysis; (e) outcomes reported; and (f) confounding factors adjusted. The quality (article selection, comparability, and outcomes) were assessed using the Newcastle–Ottawa Scale (NOS) [36].

### Statistical analyses

HRs and the 95% confidence intervals (CIs) were used as indicators regarding the correlation of TyGs with MACCEs in individuals with AMI. For cohorts with TyGs analyzed as categorical variables, HRs of MACCEs occurrences in populations with the highest TyGs versus with the lowest were collected. For cohorts with TyGs analyzed as continuous variables, HRs of MACCEs incidences per 1 unit increment of TyGs was collected. Before pooled analyses, HRs were logarithmically converted. The Cochran’s Q test was utilized to calculate the I^2^ statistics, and heterogeneity revealed statistically significant when *I*^2^ > 50. Random-effects model was applied to pool HRs. Subgroup analyses were carried out based on study characteristics, including diabetic status, treatment for coronary occlusion, and type of AMI, on the correlation of TyG with MACCEs risk. Publication bias was evaluated graphically via funnel plots. Sensitivity analysis was conducted using one-by-one elimination method. RevMan (Version 5.4) and STATA (Version 18) were applied to conduct these analyses.

## Results

### Study search

**Fig. 1:**
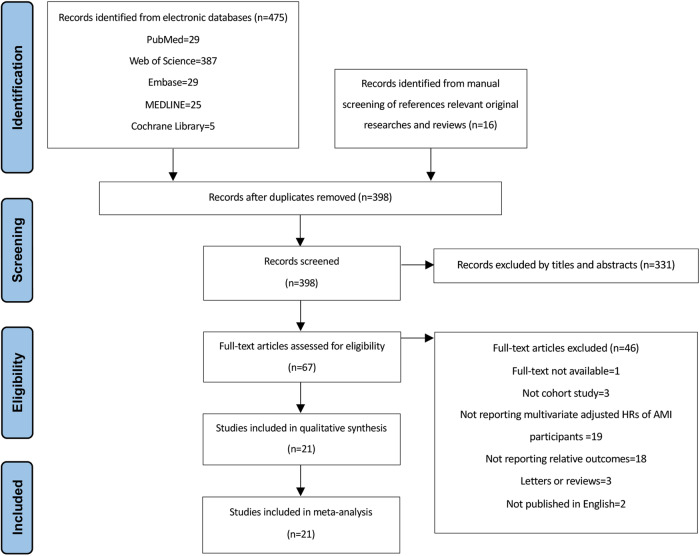
PRISMA flowchart of search strategy and study selection.

In all, 398 studies were identified after excluding duplications. Among, 67 articles underwent full-text scanning, and of those, a total of 21 cohorts were finally included [14–34] (Fig. 1).

### Study characteristics and quality assessment

Overall, 22 cohorts (16 retrospective and 5 prospective) comprising a total of 20403 participants diagnosed with AMI at baseline were included in our current review. All articles were published between 2019 and 2023. Among, majority of included studies, 19 in 21, were from China [14–24, 26–30, 32–34], while another 2 were performed in Turkey [25, 31]. Sample sizes varied from 131 to 3181, and the mean age varied from 44.5 to 76.5 years, with male proportion varying from 55.9 to 81.8% and DM proportion varying from 0 to 100%. TyG index was analysed as categorical variables in six cohorts [14, 17, 21, 30, 31, 34], as continuous variables in twelve cohorts [15, 16, 18, 20, 22–28, 32], and as both in three cohorts [19, 29, 33]. The follow-up periods varied from 12.0 to 51.7 years. Age, sex, medical history, laboratory findings, angiographic findings, and in-hospital medications were adjusted to varying degrees when the correlation of TyGs with MACCEs or all-cause death in AMIs were reported (Table 1). The NOS scores were nine for all cohorts, suggesting good quality (Table 2).

**Table 1:** Baseline characteristics of included studies.

Study, publication year	Design	follow-up duration (months)	Number of participants	AMI	Data source	Mean age (years)	Male (%)	STEMI (%)	DM (%)	TyG index analysis	Outcomes	Variables adjusted
Luo, et al. [14]	RC	12.0	1092	1092	China, Nanjing, Zhongda Hospital (2012-2018)	62.4	79.1	100	24.7	Q4:Q1	MACCEs	Age, DBP, Killip class >1, smoking, anemia, WBC, PLT, ALB, UA, eGFR, coronary angiography outcomes, and LVEF
Ma, 2020	RC	30.0	776	166	China, Beijing, Beijing Anzhen Hospital (2016-2017)	61	72.2	8.5	100	continuous	MACCEs	age, BMI, DBP, HDL-C, HbA1c, sex, smoking, drinking, PAD, CKD, HF, prior MI, prior PCI, insulin and/or oral antidiabetic agents, CAD, drug-coated balloon, lesions >20 mm long, and ICR
Wang, et al. [16]	RC	36.0	2531	513	China, Tianjin, Tianjin Chest Hospital (2016)	66.3	55.9	10.4	100	continuous	MACCEs	age, male, smoker, prior MI, prior CABG, BMI, AMI, LVEF, LM/multivessel disease, HbA1c, hs-CRP, statin, and insulin
Zhang, et al. [17]	RC	33.3	3181	3181	China, Beijing, Beijing Friendship Hospital (2013-2019)	63.3	75.7	NA	38.7	Q1:Q2	MACCEs	age, gender, DM, HP, prior AMI, HbA1c, eGFR, TGs, LVEF, and multivessel/LM
Zhao, et al. [18]	RC	36.0	798	148	China, Beijing, Beijing Anzhen Hospital (2015)	60.9	68.3	0	100	continuous	MACCEs	age, sex, BMI, SBP, DBP, smoking, drinking, DM, dyslipidemia, prior MI, PCI, stroke, PVD, NSTEMI, TC, HDL-C, eGFR, HbA1c, LVEF, bSS, LM treatment, DCB use, stents number, DAPT use, statins use, oral hypoglycemic agents, insulin, and ICR
Gao, et al. [19]	PC	41.7	1179	1179	China, Beijing, Fuwai Hospital (2015-2019)	55.7	73.5	39.2	15.8	Q1:Q3; continuous	MACCEs	age, sex, MI type, HP, DM, and dyslipidemia
Yang, et al. [20]	PC	29.0	5489	814	China, Beijing, Fuwai Hospital (2013)	57.2	79.4	NA	0	continuous	MACCEs	age, gender, dyslipidaemia, prior stroke, MI, PCI, and CABG, multivessel disease, CTO, ISR, bSS, stent number, LVEF, FBG, HbA1c, and hs-CRP
Zhang, et al. [21]	RC	26.8	1932	1932	China, Beijing, Beijing Friendship Hospital (2013-2020)	65.4	68.5	46.1	0	Q1:Q3	MACCEs; all-cause death	age, BMI, stroke, prior PCI, antiplatelet agents, WBC, Hb, ALB, eGFR, LVEF, angiography findings, in-hospital therapy, and hypoglycaemic agents
Zhao, et al. [22]	RC	48.0	1510	239	China, Beijing, Beijing Anzhen Hospital (2015)	59.7	73.7	0	0	continuous	MACCEs	age, gender, BMI, smoking, HP, dyslipidaemia, prior MI, PCI, stroke and PAD, TC, HDL-C, eGFR, HbA1c, LVEF, angiography findings, DAPT use, statins use, and ACEI/ARB use
Chen, et al. [23]	RC	24.0	1578	131	China, Beijing, Beijing Anzhen Hospital (2017-2019)	62.9	70.7	5.3	0	continuous	MACCEs	age, sex, BMI, smoking, HP, prior MI, stroke, prior PCI, HF, CKD, LVEF, LDL-C, HDL-C, CAD type, LM disease, ICR, and IABP use
Guo, et al. [24]	RC	In-hospital	1648	1648	China, Nanjing, Zhongda Hospital (2012-2018)	76.5	74.0	53.0	0	continuous	All-cause death	age, drinking, smoking, ALB, ALT, BUN, Cr, Hb, TC, SBP, UA, and WBC
Saylik, et al. [25]	RC	12.0	430	430	Turkey, Istanbul, Van Training and Research Hospital (2015-2020)	70.0	67.7	100	0	continuous	All-cause death	platelets, GRACE risk score, HR, and the presence of no-reflow
Shao, et al. [26]	PC	30.9	1694	440	China, Beijing, Beijing Anzhen Hospital (2016-2017)	60.0	76.5	12.8	45.8	continuous	MACCEs	sex, BMI, smoking, HP, DM, dyslipidemia, prior MI, prior PCI, CKD, GRACE, hs-CRP, bSS, ICR, and medications at discharge
Wu, et al. [27]	RC	68.0	526	153	China, Jinan, Qilu Hospital (2013-2018)	44.5	60.2	NA	19.6	continuous	MACCEs	age, gender, BMI, LVEF, admission for MI, multivessel disease, GS, smoking, drinking, FH-CAD, DM, HP, hyperlipidemia, TC, LDL-C, HDL-C, eGFR, UA, medications
Xiong, et al. [28]	RC	30.7	986	521	China, Sichuan, Third People’s Hospital of Chengdu (2018-2020)	66.6	71.7	30.4	34.8	continuous	MACCEs	GRACE score, female, bSS, ICR, LVEF, diuretics
Fu, et al. [29]	PC	In-hospital	2190	2190	Nationwide, multicenter CAMI registry (2014-2016)	59.6	80.6	100	35.6	Q1:Q3; continuous	MACCEs; all-cause death	age, sex, anterior MI, Killip class III/IV, primary PCI, smoking, prior stroke, CKD, HR, SBP, LVEF, Cr, LDL-C, medications
Hao, et al. [30]	RC	12.0	1144	1144	China, Nanjing, Zhongda Hospital (2018-2020)	62.1	78.8	57.2	18.9	Q1:Q3	All-cause death	age, ALB, BNP, Cr, CVD, DM, eGFR, FPG, GRACE score, HF, Hb, Killip class >1, LVEF, stent implantation, UA
Karadeniz, et al. [31]	RC	12.0	1694	1694	Turkey, Karaman, Karamanoglu Mehmetbey University (2017-2022)	64.0	70.3	38.1	28.9	Q1:Q2	MACCEs	female, age, smoking, HP, DM, CHD, hyperlipidemia, CHF, CRF, treatment for coronary occlusion, laboratory findings
Ye, et al. [32]	RC	51.7	573	573	China, Anhui and Hubei, Xuancheng Hospital and Xiangyang Central Hospital (2015-2018)	57.0	81.8	100	25.5	continuous	MACCEs	age, DM, smoking
Zhang, et al. [33]	RC	47.0	1650	674	China, Xi’an, First Affiliated Hospital of Xi’an Jiaotong University	60.5	77.5	33.7	0	Q1:Q2; continuous	MACCEs	gender, STEMI, drinking, stents number, LM disease, two/three-vessel disease, Hb, FBG, TG, and β-blocker medication
Zhao, et al. [34]	PC	23.5	1541	1541	China, Beijing, Fuwai Hospital (2017-2019)	61.0	80.3	100	34.5	Q1:Q2	All-cause death	sex, age, HP, hyperlipidemia, DM, Cr, TIMI, IABP, prior CABG, prior PCI, CKD, LDL-C, hs-CRP, height, weight, smoking, DBP, SBP, HR, and statin use

*AMI* acute myocardial infarction, *STEMI* ST-segment elevation myocardial infarction, *DM* diabetes mellitus, *TyG* triglyceride–glucose, *RC* retrospective cohort, *PC* prospective cohort, *MACCEs* major adverse cardiovascular and cerebrovascular events, *DBP* diastolic blood pressure, *SBP* systolic blood pressure, *WBC* white blood cells, *PLT* Platelets, *ALB* albumin, *UA* uric acid, *eGFR* estimated glomerular filtration rate, *LVEF* left ventricular ejection fraction, *BMI* body mass index, HDL-C high-density lipoprotein cholesterol, HbA1c glycosylated hemoglobin, PAD peripheral vascular disease, CKD chronic kidney disease, HF heart failure, *CAD* coronary artery disease, *PCI* percutaneous coronary intervention, *ICR* complete revascularization, *LM* left main, *TC* total cholesterol, *TG* triglyceride, *DAPT* dual anti-platelet therapy, *DCB* drug coated balloon, *HP* hypertension, *LDL-C* low-density lipoprotein cholesterol, *IABP* intra-aortic balloon pump, *BNP* brain natriuretic peptide, *CTO* chronic total occlusion.

**Table 2:** Newcastle–Ottawa Scale appraisal of included studies.

Study	Representativeness of the exposed cohort	Selection of the non-exposed cohort	Ascertainment of exposure	Outcome not presenting at baseline	Control for age	Control for other confounding factors	Assessment of outcome	Significant follow-up duration	Adequacy of follow-up of cohorts	Total
Luo, et al. [14]	1	1	1	1	1	1	1	1	1	9
Ma, 2020	1	1	1	1	1	1	1	1	1	9
Wang, et al. [16]	1	1	1	1	1	1	1	1	1	9
Zhang, et al. [17]	1	1	1	1	1	1	1	1	1	9
Zhao, et al. [18]	1	1	1	1	1	1	1	1	1	9
Gao, et al. [19]	1	1	1	1	1	1	1	1	1	9
Yang, et al. [20]	1	1	1	1	1	1	1	1	1	9
Zhang, et al. [21]	1	1	1	1	1	1	1	1	1	9
Zhao, et al. [22]	1	1	1	1	1	1	1	1	1	9
Chen, et al. [23]	1	1	1	1	1	1	1	1	1	9
Guo, et al. [24]	1	1	1	1	1	1	1	1	1	9
Saylik, et al. [25]	1	1	1	1	1	1	1	1	1	9
Shao, et al. [26]	1	1	1	1	1	1	1	1	1	9
Wu, et al. [27]	1	1	1	1	1	1	1	1	1	9
Xiong, et al. [28]	1	1	1	1	1	1	1	1	1	9
Fu, et al. [29]	1	1	1	1	1	1	1	1	1	9
Hao, et al. [30]	1	1	1	1	1	1	1	1	1	9
Karadeniz, et al. [31]	1	1	1	1	1	1	1	1	1	9
Ye, et al. [32]	1	1	1	1	1	1	1	1	1	9
Zhang, et al. [33]	1	1	1	1	1	1	1	1	1	9
Zhao, et al. [34]	1	1	1	1	1	1	1	1	1	9

### TyG index and the occurrence of MACCEs

**Fig. 2:**
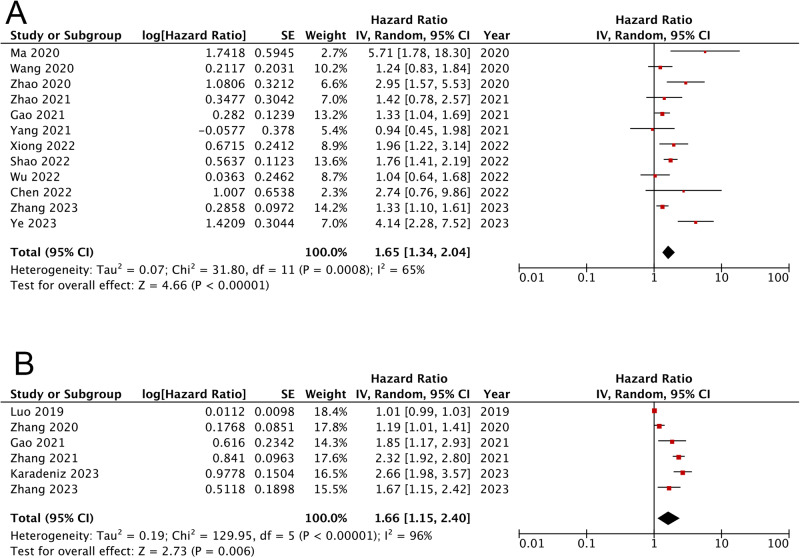
Forest plots for the meta-analysis of the association between the TyG index and the risk of composite MACCEs. **A** Meta-analysis with the TyG index analysed as a continuous variable. **B** Meta-analysis with the TyG index analysed as a categorical variable.

**Fig. 3:**
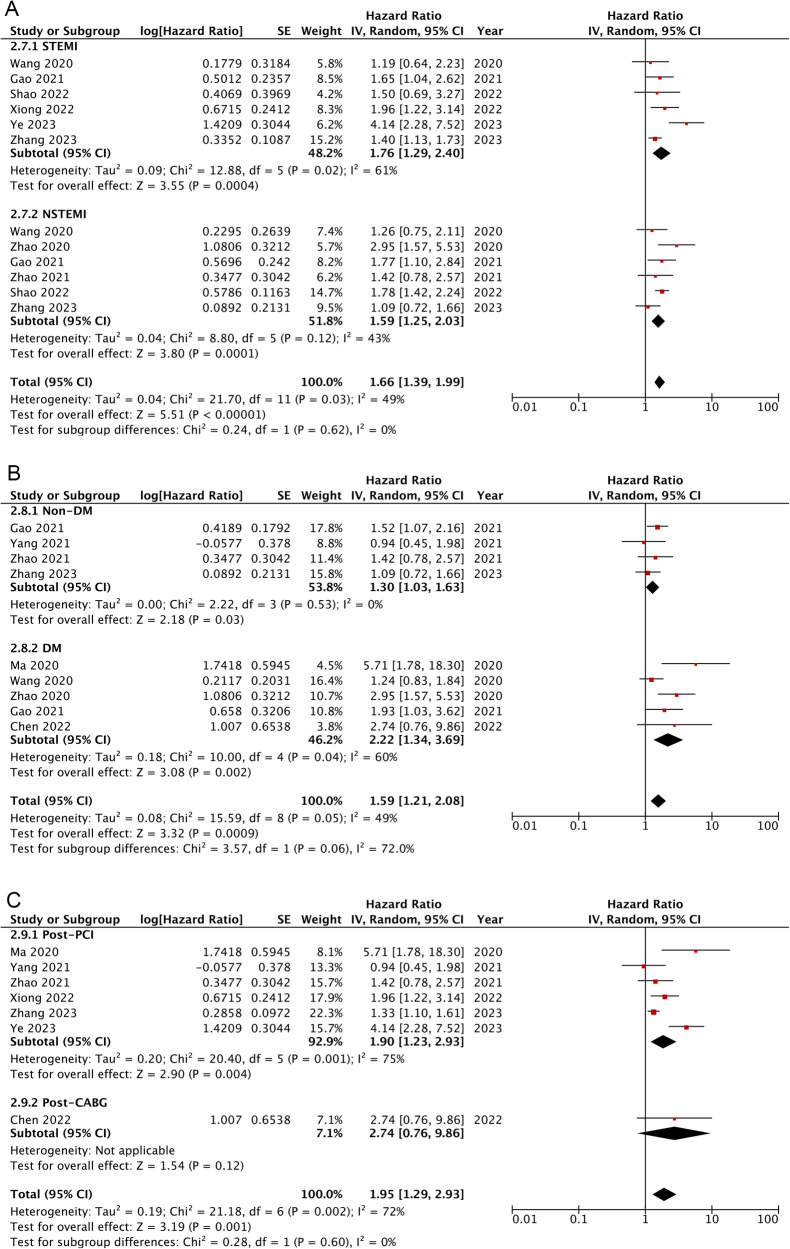
Subgroup analyses for the association between the TyG index analysed as a continuous variable and the risk of composite MACCEs. **A** Subgroup analysis according to the type of AMI. **B** Subgroup analysis according to the diabetic status. **C** Subgroup analysis according to the reperfusion strategy.

Overall, findings from twelve cohorts demonstrated that in comparison to AMI patients categorized with the lowest TyG [15, 16, 18–20, 22, 23, 26–28, 32, 33], those in the highest category showed apparently increased risks of MACCEs (HR: 1.65, 95% CI: 1.34-2.04, *I*^2^ = 65%, *P* < 0.00001) (Fig. 2A). Similar results were showed as TyG index analysed as continuous variables [14, 17, 19, 21, 31, 33] (HR: 1.66, 95% CI: 1.15–2.40, *I*^2^ = 96%, *P* = 0.006) (Fig. 2B). Subgroup analyses presented that AMI populations with higher TyG exhibited apparently increased risks of MACCEs independent of diabetic status, type of AMI, and treatment for coronary occlusion (for subgroups, all *P* < 0.05) (Fig. 3A–C). Sensitivity analysis suggested that the association of TyG index with MACCEs was robust (Supplementary Fig. 1A, B).

### TyG index and the occurrence of all-cause death

**Fig. 4:**
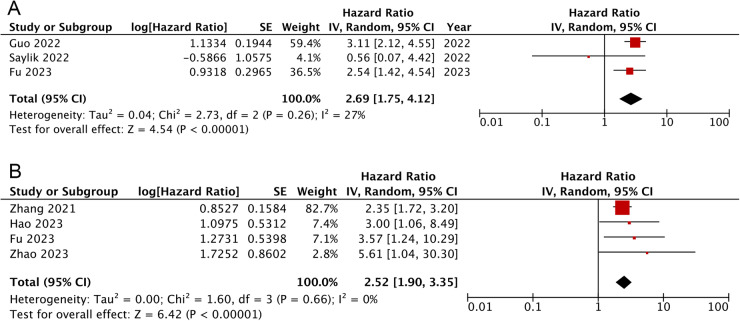
Forest plots for the meta-analysis of the association between the TyG index and the risk of all-cause death. **A** Meta-analysis with the TyG index analysed as a continuous variable. **B** Meta-analysis with the TyG index analysed as a categorical variable.

Overall, results of three cohorts exhibited that in comparison to AMI populations in the lowest TyG category [24, 25, 29], those in the highest category showed significantly increased risk for all-cause death (HR: 2.69, 95% CI: 1.75–4.12, *I*^2^ = 27%, *P* < 0.00001) (Fig. 4A). These findings were in line with results when TyG index analysed as continuous variables [21, 29, 30, 34] (HR: 2.52, 95% CI: 1.90-3.35, *I*^2^ = 0%, *P* < 0.00001) (Fig. 4B). Sensitivity analysis indicated that the correlation of TyG with all-cause death was robust when TyG analysed as continuous variables (Supplementary Fig. 1C, D).

### Publication bias

**Fig. 5:**
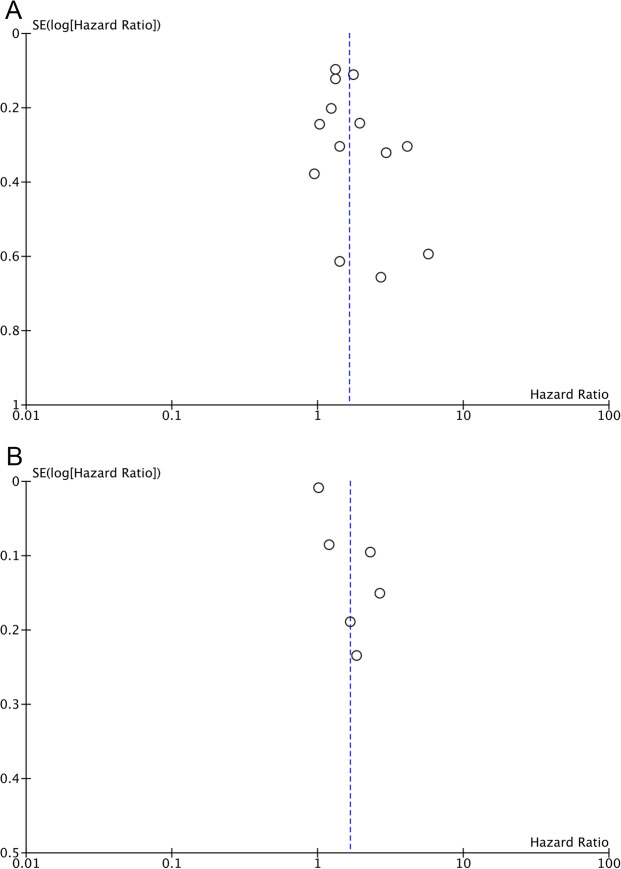
Funnel plots for the publication bias underlying the meta-analysis of the association between the TyG index and the risk of composite MACCEs. **A** Funnel plots underlying the meta-analysis with TyG index analysed as a continuous variable. **B** Funnel plots underlying the meta-analysis with TyG index analysed as a categorical variable.

Funnel plots describing the correlation of TyG analysed as categorical and continuous variables with MACCEs were listed in Fig. 5. No significant asymmetry of the funnel plots was observed, suggesting a minimal likelihood of biased publication.

## Discussion

In this pooled analysis, we showed that patients with higher TyG index exhibited apparently increased incidences of both MACCEs and all-cause death. For composite MACCEs, subgroup analyses indicated that this relationship was in a stable state as not affected by diabetic status, type of AMI, nor reperfusion treatment of the patients. All findings pointed that higher TyG index could be a potential indicator for elevated risks of MACCEs, as well regarding all-cause death in AMI population.

All along, the hyper insulinemic euglycemic clamp technique is deemed as the “gold criterion” for measurement of insulin sensitivity [37]; nevertheless, the use of this technique was restricted due to its time-consuming, expensive and complicated characteristics [38]. Recently, some other standards effectively diagnosing IR have been gradually developed, for instance, HOMA-IR, QUICKI, and TG/HDL-C [10, 12, 39], thereinto, also including the TyG index that our meta-analysis focused [40]. Current findings confirmed the potential use of the TyG index as a dependable marker for IR. A previous study confirmed both higher sensitivity and specificity using TyG index to detect IR, in comparison to hyper-insulinemic-euglycemic clamp test [41]. Furthermore, evidence explored by Tam et al. also showed a better performance of TyG index in assessing IR than homeostasis model assessment [42]. Methodologically, considering its easily accessible, relevantly safe, and comparatively cheap advantages, we deemed TyG as a not only reliable but also practical index to assess IR in patients with AMI, for often urgent conditions the AMI population placed and thus no ability to satisfy some complex exam procedures.

This is the first systematic review to pool the association of TyG index with the risks of subsequent incidences of MACCEs and all-cause death in AMI population. A previous meta-analysis has illuminated the correlation between TyG index and outcomes in individuals with coronary artery disease [43]. Recent meta-analyses have also identified a positive link between TyG index and the occurrence of atherosclerotic cardiovascular diseases in general population [44, 45]. In comparison to these previous meta-analyses, our study specifically focused on individuals with AMI to provide more precise results for this specific population. Furthermore, the correlation between TyG index and other metabolic cardiovascular disorders, for instance, heart failure [46], coronary artery calcification [47], and arterial stiffness [47, 48], has been previously established. Our study supported the potential value of TyG index to be applied as a predictor of MACCEs risk in AMI patients, by which we were not surprised. Currently, IR has been confirmed that was associated with the worse outcomes following CABG [49]. Then, pathologically, IR might directly result in endothelial dysfunctions [50, 51], hence, aggravating coronary cramp and consequently increasing the occurrence of no-reflow events or microcirculation disturbance after coronary reperfusion, which contributed to pushing ischemia myocardium to the infarcted state and subsequent an intensified infarct size [52–55]. Moreover, IR has been indicated related to disorders of sympathetic nervous system [56] and imprisons of cardiac autonomic function [57], that would facilitate adverse clinical outcomes during AMI such as malignant arrhythmia, cardiac arrest, sudden death, etc. [58, 59]. In this study, only cohorts were included, thereby effectively avoiding potential recall biases related to cross-sectional design. Furthermore, all the outcomes considered in this analysis were meticulously adjusted for multiple variables. Additionally, subgroup analyses were conducted to ensure the robustness of these findings and ascertain that they were not influenced by patient characteristics such as diabetic status, AMI type, or reperfusion therapy. Nevertheless, further investigations are warranted to determine whether incorporating TyG into existing prediction tools can enhance predictive efficacy for clinical outcomes among individuals with AMI.

There are still some limitations in our meta-analysis. First, there were few studies accessible for this study, and there was substantial heterogeneity among them, which possibly due to variations in the methods of measuring the TyG index, study design, follow-up duration and AMI type among the studies. Further research is necessary to investigate whether different study characteristics such as participant ethnicity, follow-up duration, and concurrent medications could impact these findings. Second, most included studies originated from China, with only 2 out of 21 conducted in European regions. This might limit the generalizability of the predictive value of TyG index for outcomes in AMI populations universally. Third, all research included were cohort studies, thus, causal link between TyG and MACCEs could not be definitively established. Finally, events such as inter-institutional differences in in-hospital management might have prognostic implications in patients with AMI that might weaken the meaningfulness of conclusions in this review to some extent.

## Conclusion

In conclusion, based on existing findings from this meta-analysis, higher TyG index might be a reliable indicator for MACCEs in individuals suffering from AMI. Further explorations are imperative and essential to ascertain whether the integration of the TyG index in conjunction with current prediction tools for AMI could enhance their prognostic capabilities.

### Supplementary information

supplementary Figure 1

Table S1

Supplementary Material Legends

ICMJE conflicts of interest form

## Data Availability

All data generated can be obtained from this published article and its additional information files.
