# Supplementary figures and images for: The association of triglyceride–glucose index with major adverse cardiovascular and cerebrovascular events after acute myocardial infarction: a meta-analysis of cohort studies
Source: Nutr Diabetes. 2024 Jun 6;14:39. doi: 10.1038/s41387-024-00295-1 (PMC11156940; doi:10.1038/s41387-024-00295-1)

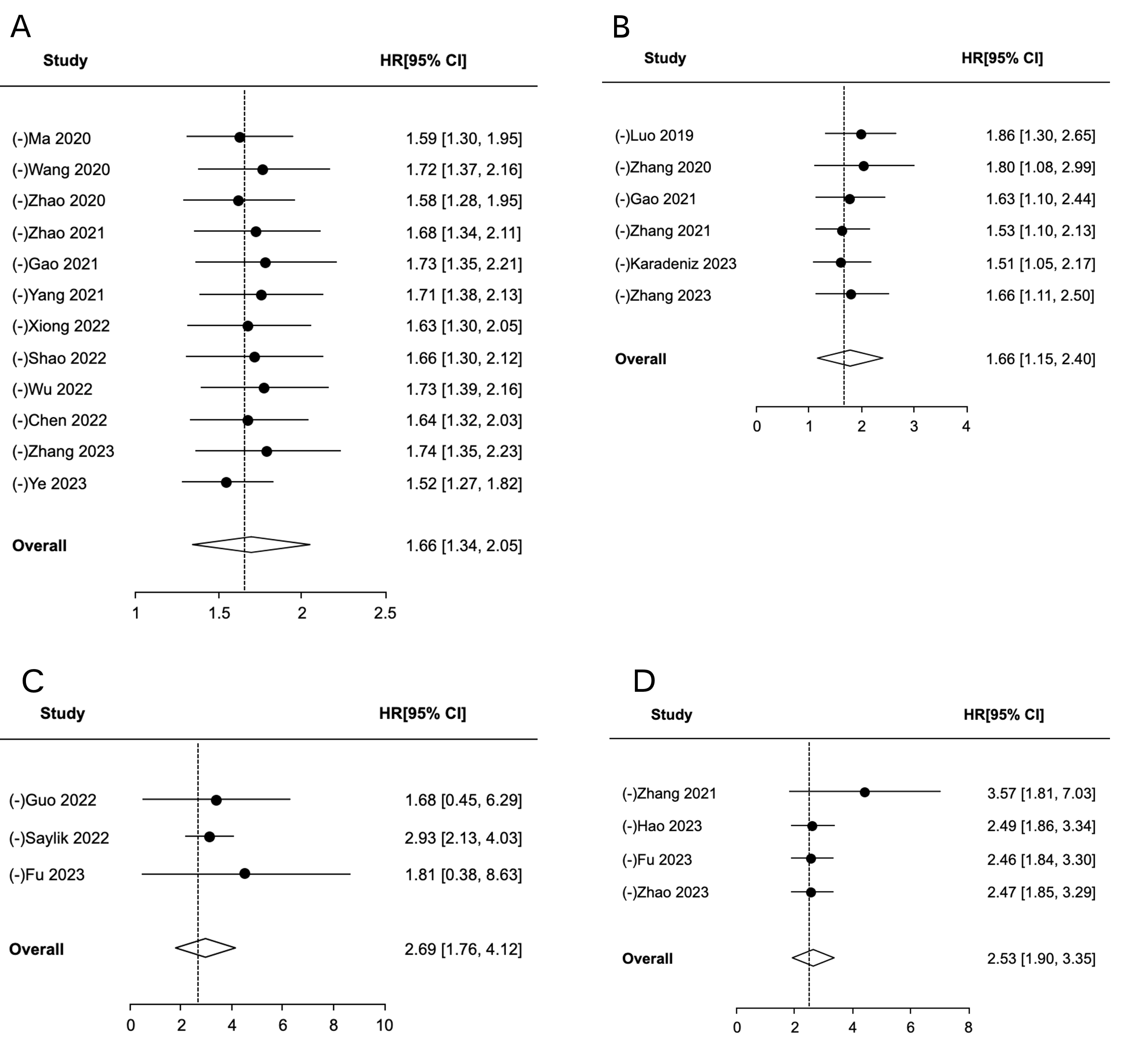

Supplement: Supplementary file 1 — supplementary Figure 1 [file 41387_2024_295_MOESM1_ESM.tif]
