# Supplementary material for: The association of triglyceride–glucose index with major adverse cardiovascular and cerebrovascular events after acute myocardial infarction: a meta-analysis of cohort studies
Source: Nutr Diabetes. 2024 Jun 6;14:39. doi: 10.1038/s41387-024-00295-1 (PMC11156940; doi:10.1038/s41387-024-00295-1)

**Table S1**

**PubMed 29 2023.10.05**

**(((TyG index[Title/Abstract]) OR (triglyceride-glucose index[Title/Abstract])) OR (triglyceride glucose index[Title/Abstract])) AND (((((((((((((((((((("Anterior Wall Myocardial Infarction"[Title/Abstract]) OR ("Inferior Wall Myocardial Infarction"[Title/Abstract])) OR (MINOCA[Title/Abstract])) OR ("Non-ST Elevated Myocardial Infarction"[Title/Abstract])) OR ("Shock, Cardiogenic"[Title/Abstract])) OR ("ST Elevation Myocardial Infarction"[Title/Abstract])) OR ("Infarction, Myocardial"[Title/Abstract])) OR ("Infarctions, Myocardial"[Title/Abstract])) OR ("Myocardial Infarctions"[Title/Abstract])) OR ("Cardiovascular Stroke"[Title/Abstract])) OR ("Cardiovascular Strokes"[Title/Abstract])) OR ("Stroke, Cardiovascular"[Title/Abstract])) OR ("Strokes, Cardiovascular"[Title/Abstract])) OR ("Myocardial Infarct"[Title/Abstract])) OR ("Infarct, Myocardial"[Title/Abstract])) OR ("Infarcts, Myocardial"[Title/Abstract])) OR ("Myocardial Infarcts"[Title/Abstract])) OR ("Heart Attack"[Title/Abstract])) OR ("Heart Attacks"[Title/Abstract])) OR ("Myocardial Infarction"[Mesh]))**

**Embase 29**

**('triglyceride-glucose index'/exp OR 'triglyceride-glucose index':ab OR 'TyG index':ab OR 'TyG index'/exp OR 'triglyceride glucose index':ab OR 'triglyceride glucose index'/exp) AND ('acute heart infarction'/exp OR 'infarction, myocardial':ab OR 'infarctions, myocardial':ab OR 'acute myocardial infarctions':ab OR 'acute cardiovascular stroke':ab OR 'acute cardiovascular strokes':ab OR 'stroke, cardiovascular':ab OR 'strokes, cardiovascular':ab OR 'acute myocardial infarct':ab OR 'infarct, myocardial':ab OR 'infarcts, myocardial':ab OR 'acute myocardial infarcts':ab OR 'acute heart attack':ab OR 'acute heart attacks':ab OR 'anterior wall myocardial infarction':ab OR 'inferior wall myocardial infarction':ab OR 'minoca':ab OR 'non-st elevated myocardial infarction':ab OR 'shock, cardiogenic':ab OR 'st elevation myocardial infarction':ab)**

**Web of science 387**

# 1 (TS=(**'triglyceride-glucose index' OR 'TyG index' OR 'triglyceride glucose index'**)) OR (AB=(**'triglyceride-glucose index' OR 'TyG index' OR 'triglyceride glucose index'**))

**#2 (AB=('acute heart infarction' OR 'infarction, myocardial' OR 'infarctions, myocardial' OR 'acute myocardial infarctions' OR 'acute cardiovascular stroke' OR 'acute cardiovascular strokes' OR 'stroke, cardiovascular' OR 'strokes, cardiovascular' OR 'acute myocardial infarct' OR 'infarct, myocardial' OR 'infarcts, myocardial' OR 'acute myocardial infarcts' OR 'acute heart attack' OR 'acute heart attacks' OR 'anterior wall myocardial infarction' OR 'inferior wall myocardial infarction' OR 'minoca' OR 'non-st elevated myocardial infarction' OR 'shock, cardiogenic' OR 'st elevation myocardial infarction')) OR (TS=('acute heart infarction'))**

**#1 AND #2**

**MEDLINE 25**

**MESH subheading, Title, Abstract**

**'Triglyceride-glucose index' OR 'TyG index' OR 'triglyceride glucose index'**

**'acute heart infarction' OR 'infarction, myocardial' OR 'infarctions, myocardial' OR 'acute myocardial infarctions' OR 'acute cardiovascular stroke' OR 'acute cardiovascular strokes' OR 'stroke, cardiovascular' OR 'strokes, cardiovascular' OR 'acute myocardial infarct' OR 'infarct, myocardial' OR 'infarcts, myocardial' OR 'acute myocardial infarcts' OR 'acute heart attack' OR 'acute heart attacks' OR 'anterior wall myocardial infarction' OR 'inferior wall myocardial infarction' OR 'minoca' OR 'non-st elevated myocardial infarction' OR 'shock, cardiogenic' OR 'st elevation myocardial infarction' OR 'acute myocardial infarction'**

**Cochrane library 5**


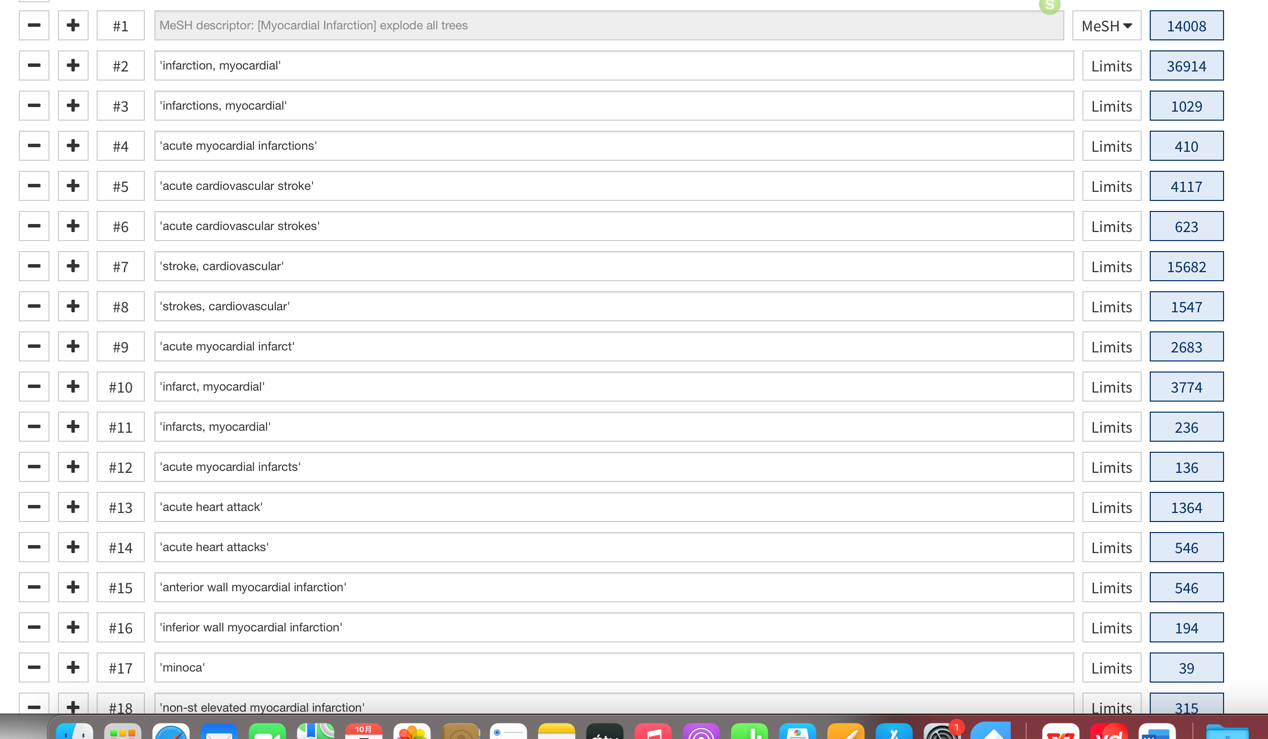


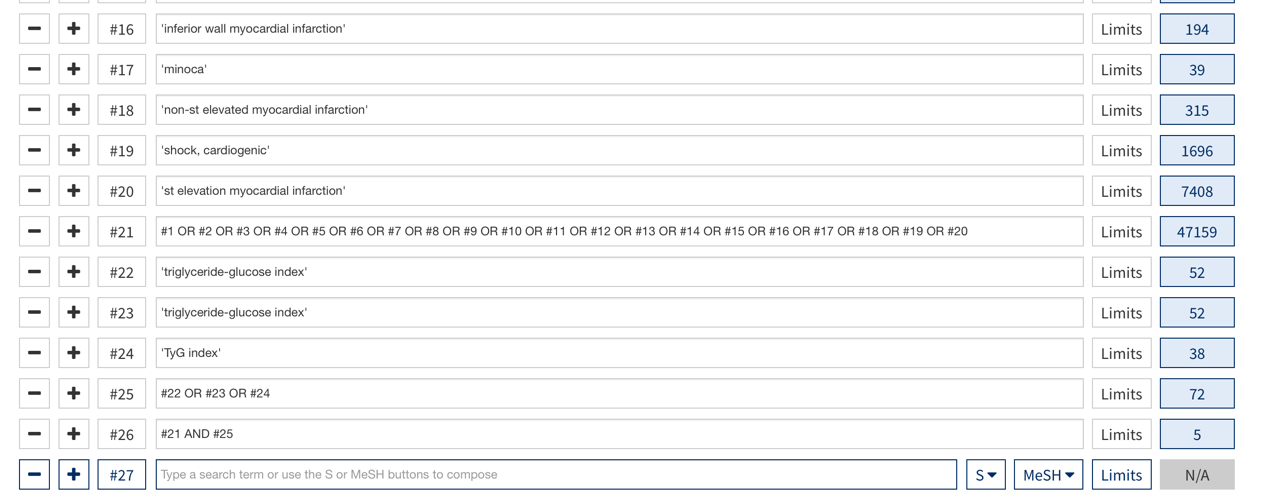

Supplement: Supplementary file 2 — Table S1 [file 41387_2024_295_MOESM2_ESM.docx]
