# Supplementary material for: The association of triglyceride–glucose index with major adverse cardiovascular and cerebrovascular events after acute myocardial infarction: a meta-analysis of cohort studies
Source: Nutr Diabetes. 2024 Jun 6;14:39. doi: 10.1038/s41387-024-00295-1 (PMC11156940; doi:10.1038/s41387-024-00295-1)
Supplement: Supplementary file 3 — Supplementary Material Legends [file 41387_2024_295_MOESM3_ESM.docx]

**Supplementary Material Legends**

**Supplementary Figure 1.** Sensitivity analyses for the association of the TyG index analysed as categorical (A) or continuous variables (B) with the risk of MACCEs, and the association of the TyG index analysed as categorical (C) or continuous variables (D) with the risk of all-cause death.

**Supplementary Table 1.** Full search strategy.
